# Retroperitoneal perforation arising from duodenal diverticulum treated by endoscopic drainage: a case report

**DOI:** 10.1002/ccr3.921

**Published:** 2017-03-29

**Authors:** Takashi Shirobe, Hideyuki Kawakami, Sadanori Abe, Tomoki Yokochi

**Affiliations:** ^1^ Department of SurgeryNarita‐Tomisato Tokushukai HospitalTomisatoChibaJapan; ^2^ Department of Clinical ResearchChiba Tokushukai HospitalFunabashiChibaJapan

**Keywords:** Duodenal diverticulum, duodenal perforation, endoscopic drainage, retroperitoneal abscess, retroperitoneal perforation

## Abstract

Retroperitoneal perforation of duodenal diverticula around the papilla of Vater is relatively rare. In this report, we describe retroperitoneal abscess, which was successfully treated by endoscopic drainage. Thus, endoscopic approach for retroperitoneal perforation caused by diverticulum is one of the treatment options in addition to surgery.

## Introduction

The duodenum is the most frequent site of diverticulum next to the large bowel 1, 2. Duodenal diverticulum around the ampulla of Vater is referred to as juxta‐papillary diverticulum. Perforation of diverticulum at the large intestine is quite common, requiring surgical treatment for panperitonitis or retroperitoneal abscess. On the other hand, perforation of diverticulum at the duodenum is relatively rare. Duodenal diverticulum is one of the causes of obstruction to the outflow of pancreatic juice or bile, resulted in Lemmel's syndrome. In this case report, we describe the patient with retroperitoneal abscess caused by perforation of juxta‐papillary diverticulum.

## Case Report

**Figure 1:**
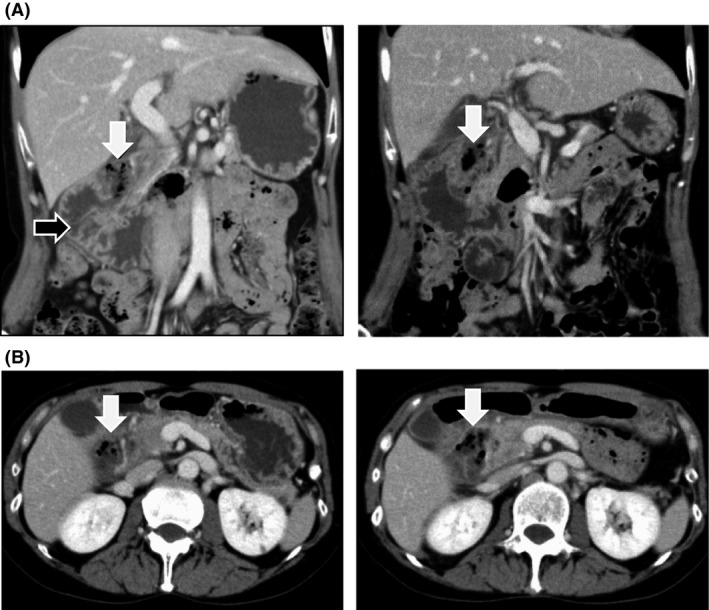
Coronal (A) and axial (B) section images by CT scans on the day of admission (the 1st day, left panels) and the 3rd day (right panels) showed an increase of retroperitoneal abscess (white arrowheads) apparently connected to juxta‐papillary diverticulum. The dilation and wall thickening of the descending part of duodenum is also indicated (black arrowhead).

A 52‐year‐old woman was admitted to our hospital due to sudden onset of pain at right hypochondriac region. The patient had undergone total mastectomy for left breast cancer 10 years ago. Results of blood tests were normal except increased number of white blood cells (WBC) (12,900/mm^3^). CRP was within normal range (0.11 mg/dL). Computed tomography (CT) scans showed significant dilation of the descending part of duodenum, mural edema, and accumulation of fluid and air (Fig. 1). As abdominal CT suggested retroperitoneal abscess (40 × 27 mm) caused by perforation at juxta‐papillary duodenal diverticulum, the patient was immediately hospitalized and was treated with conservative therapy consisting of nil per oral, parenteral nutrition, and a course of antibiotics (a meropenem dose of 0.5 g intravenously, every 12 h for 8 days).

**Figure 2:**
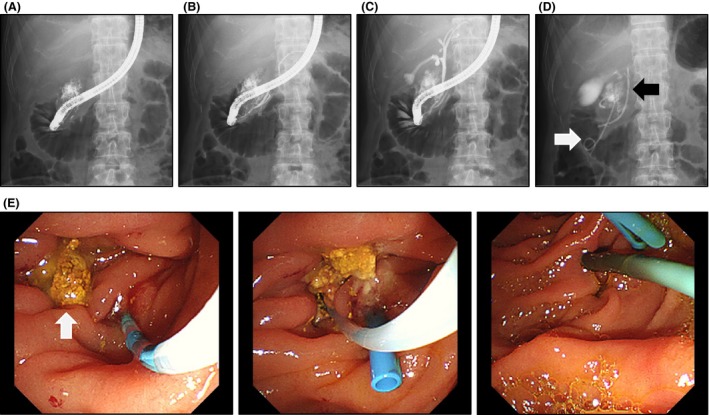
Endoscopic contrast study of duodenal diverticulum on the 3rd day from admission showed retroperitoneal abscess (A). Endoscopic retrograde pancreatography yielded normal results at the main pancreatic duct (B). Endoscopic cholangiography apparently indicated lower part of the bile duct excluded by abscess (C). Stents were placed in the bile duct (black arrowhead) and in the retroperitoneal abscess cavity (white arrowhead) (D). (E) Inner views during the endoscopic procedure on the 3rd day (left and middle panels) and 20th day (right panel) were also shown. Note that a bilirubin calculus found on the 3rd day (indicated by white arrowhead) had dropped on the 20th day.

**Figure 3:**
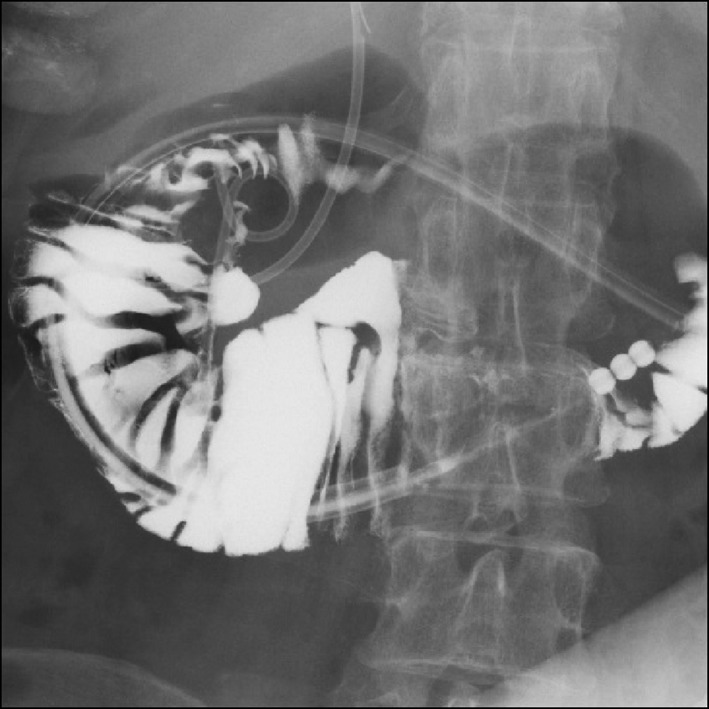
Hypotonic duodenography conducted on the 15th day from admission indicated that abscess cavity was diminished, while least leakage of contrast media along the stent from the juxta‐papillary diverticulum was detected.

**Figure 4:**
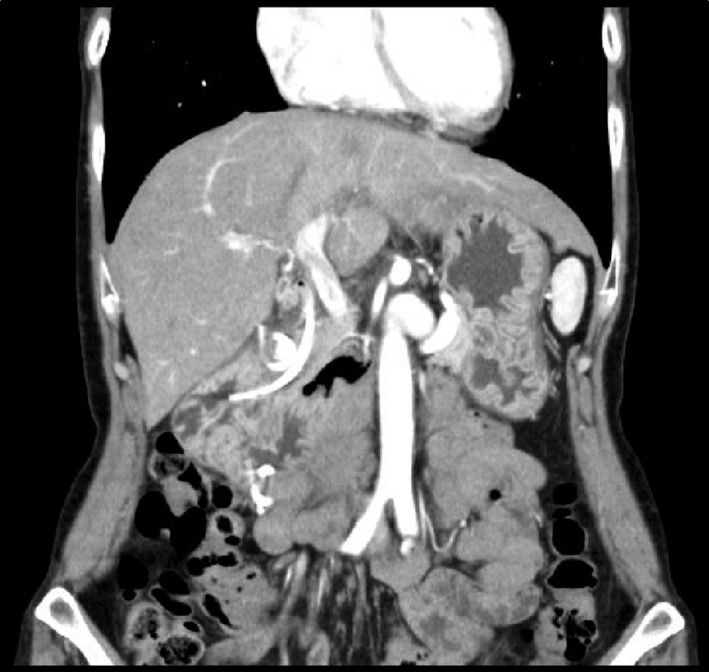
Coronal section images on the 20th day indicated that retroperitoneal abscess nearly disappeared.

In the night of the first day of admission, the patient's body temperature rose to 39.2°C. On the second day, WBC was 16,600/mm^3^ and CRP was 16.92 mg/dL, indicating worsening of inflammatory response. On the third day, contrast‐enhanced CT clearly showed an increasing retroperitoneal abscess (Fig. 1A and B). Emergency endoscopy revealed a bilirubin calculus in juxta‐papillary duodenal diverticulum and significant mucosal edema (Fig. 2). Leakage of the contrast media into the retroperitoneum was detected, suggesting retroperitoneal perforation of juxta‐papillary duodenal diverticulum (Fig. 2A). While the result of endoscopic retrograde pancreatography was normal (Fig. 2B), cholangiography indicated that the abscess was located near the bile duct (Fig. 2C). First, we placed a stent in the bile duct (disposable V‐system stent, PBD‐1033‐0705, Olympus Medical Systems, Tokyo), as we considered cholestasis caused by spreading inflammation (Fig. 2D and E). Then, we performed endoscopic placement of the next stent (Advanix double pigtail stent 18207106‐7Fr x 7 cm, Boston Scientific, MA) in the retroperitoneal abscess through the diverticulum, which was necessary for drainage of the retroperitoneal cavity (Fig. 2E). On the fourth day, the patient's body temperature fell to 37.2°C. The patient was relived from pain in just 1 day after stent implantation. CT imaging examination indicated significant reduction in the size of abscess, and oral rehydration began on the 9th day. Hypotonic duodenography conducted on the 15th day from admission indicated that retroperitoneal abscess nearly disappeared and thus the patient started a diet (Fig. 3). No post‐treatment complications occurred and CT imaging did not reveal any symptoms of recurrence on the 20th day (Fig. 4). The patient was discharged on the 22nd day. On the 40th day from admission, the stents at the bile duct and abscess cavity were removed on an outpatient basis.

## Discussion

The duodenum is the most common site for gastrointestinal diverticula after the colon 1, 2. In particular, juxta‐papillary diverticulum is frequently observed. Lemmel's syndrome is a common complication caused by juxta‐papillary diverticulum, which induces cholangitis or pancreatitis due to outflow obstruction. Unlike colon diverticulum, duodenal diverticulum is relatively asymptomatic. However, risk of perforation should be kept in mind 3. Duodenal perforation may cause retroperitoneal abscess rather than panperitonitis. Several reports described that preoperational diagnosis was difficult when emergency surgery was chosen to treat with perforated duodenal diverticulum 2, 4, 5, 6. Helical CT, however, could be employed to detect retroperitoneal air surrounding the duodenum, which may suggest perforation of juxta‐papillary duodenal diverticulum 7.

When the duodenal diverticulum perforated at the retroperitoneum, simple closure of the site is anatomically difficult. In order to excise the duodenum containing perforation, it is necessary to perform pancreaticoduodenectomy. It seems, however, too radical. Indeed, several cases reported in which surgical treatment was chosen employed only drainage for retroperitoneal perforation 8, 9. In addition, these cases took longer time to achieve postoperative cure. Alternatively, there are several literatures describing that conservative therapy utilizing antimicrobial agents, antacids, nil per oral, and parenteral nutrition was effective to cure perforated duodenal diverticulum 5, 6, 10. It should be noted that there has been reports of death, and therefore, surgical treatment in a timely manner is crucial 5, 11, 12. Given these facts, nonsurgical drainage from retroperitoneal abscess could be one of the options to treat with perforated diverticulum. As percutaneous drainage is technically difficult, endoscopic drainage is a valuable option if it provides symptomatic improvement.

In the case described in this report, a stent implantation into retroperitoneal abscess through diverticulum was significantly effective, as an immediate improvement in symptoms and decline in fever were achieved. Nonetheless, it was not easy for us to determine the best timing to restart the diet. We allowed the patient to take a food after examining the results of hypotonic duodenography on the 15th day from drainage. A previous literature reported an endoscopic approach for an abscess drainage utilizing ENBD tubes 13. In that paper, authors described that the stent tube was left in place for 6 days and food deprivation for 7 days.

We placed two stents during a single treatment, because we concerned bile duct stenosis, which was induced by progression of retroperitoneal abscess. However, there has been no literature reporting jaundice occurred by perforated retroperitoneal diverticulum. Thus, the stent placed in the bile duct may be unnecessary.

In this report, a rare case of retroperitoneal perforation at duodenal diverticulum around the ampulla of Vater was described. Endoscopic stent placement was successful, and thus, surgery was not necessary. When diffuse peritonitis is suspected as a complication of perforated diverticulum, surgical treatment is definitely the first line. However, for the patient in which perforation is strictly limited to retroperitoneal abscess, endoscopic approach has a significant advantage due to less invasiveness. Although we had only a single case, it is worth considering an endoscopic drainage of retroperitoneal abscess caused by perforation at juxta‐papillary diverticulum as one of the treatment options.

## Authorship

ST: participated in study conception and clinical design. ST, HK, and SA: involved in acquisition of clinical data. ST and TY: analyzed and interpreted the data. ST and TY: drafted and reviewed the manuscript. All authors: discussed the results and were provided the opportunity to comment on the manuscript. All authors: have read and approved the submission of this manuscript to the journal.

## Conflicts of Interest

There is no conflict of interests to be declared by authors.
